# Oral Cavity and Pharynx Cancer Incidence Trends by Subsite in the United States: Changing Gender Patterns

**DOI:** 10.1155/2012/649498

**Published:** 2012-04-17

**Authors:** Linda Morris Brown, David P. Check, Susan S. Devesa

**Affiliations:** ^1^Statistics and Epidemiology Group, RTI International, 6110 Executive Boulevard, Suite 902, Rockville, MD 20852-3907, USA; ^2^Division of Cancer Epidemiology and Genetics, National Cancer Institute, National Institutes of Health, Department of Health and Human Services, Bethesda, MD 20892, USA

## Abstract

*Objective*. To evaluate oral cavity and pharynx cancer (OCPC) patterns by gender. *Methods*. We used Surveillance, Epidemiology, and End Results program data for 71,446 cases diagnosed during 1975–2008 to classify OCPC by anatomic subsite as potentially HPV-related or not, with oral tongue cancer considered a separate category. *Results*. Total OCPC rates among men were 2–4 times those among women. Among whites, total OCPC rates rose in the younger age groups due to substantial increases in successive birth cohorts for HPV-related cancers, more rapid among men than women, and oral tongue cancers, more rapid among women than men. Among blacks, total OCPC rates declined among cohorts born since 1930 reflecting the strong downward trends for HPV-unrelated sites. Among Hispanics and Asians, HPV-unrelated cancer rates generally declined, and oral tongue cancer rates appeared to be converging among young men and women. *Conclusions*. Decreases in total OCPC incidence reflect reductions in smoking and alcohol drinking. Rising HPV-related cancers among white men may reflect changing sexual practices. Reasons for the increasing young oral tongue cancer rates are unknown, but the narrowing of the gender differences provides a clue.

## 1. Introduction

In the United States, incidence rates of oral cavity and pharynx cancer (OCPC) have been decreasing at about 1% per year over the past decade, with some differences according to race, sex, and subsite [1, 2]. Tobacco use is the major risk factor for these cancers; heavy alcohol drinking is an independent risk factor and appears to enhance the effect of smoking [3]. The attributable risk for tobacco and alcohol use has been estimated at 80% to 90% among US white and black men [3]. Dietary factors, particularly consumption of fruits and vegetables, have been consistently associated with reduced risks of OCPC [4]. Oral human papillomavirus (HPV), particularly HPV 16, is a newly recognized cause of a subset of OCPC, responsible for 40–80% of oropharyngeal squamous cell cancers [5–7]. Rising incidence rates for certain subsites within the oral cavity and pharynx, notably the base of tongue, tonsil, and oropharynx related to HPV infection, have been reported in the US particularly among young white men using data from the Surveillance, Epidemiology, and End Results (SEER) program [7–14] and National Program of Cancer Registries [15]. Rising incidence rates of oral tongue cancer, a subsite not related to HPV infection, also have been reported, especially among young white women [16]. To better understand the US incidence patterns, especially differences by gender, our analysis updates and extends earlier descriptive studies [11, 12] that classified OCPC by anatomic subsite into presumed HPV-related or HPV-unrelated oral cavity and pharynx cancers by incorporating cases diagnosed through 2008, considering oral tongue cancer not related to HPV as a separate category, including data for Hispanic whites, Asian/Pacific Islanders (Asian/PIs), and American Indian/Alaskan Natives (AI/ANs) as well as blacks and non-Hispanic whites, and presenting age-specific male/female ratios by year of birth.

## 2. Methods

Data from population-based registries in the National Cancer Institute's Surveillance, Epidemiology, and End Results (SEER) program [17, 18] were used to calculate incidence rates of invasive squamous cell carcinoma (SCC) (International Classification of Diseases for Oncology, 3rd edition (ICD-O-3) morphology codes 8050-8084) of the oral cavity (ICD-O-3 topography codes C01.9-C06.9) and pharynx (codes C09.0-C10.9, C12.9-C14.8) (excluding lip, salivary glands, and nasopharynx) [19]. Like the classification based on anatomic subsite and etiologic relationship with HPV used in two recent publications [11, 12], we categorized OCPC subsites as presumed HPV-related or not and then considered HPV-unrelated oral tongue separately. Specifically, the HPV-related sites were C01.9-base of tongue, NOS (not otherwise specified); C02.4-lingual tonsil; C09.0-tonsillar fossa; C09.1-tonsillar pillar; C09.8-overlapping lesion of tonsil; C09.9-tonsil, NOS; C10.0-vallecula; C10.1-anterior surface of epiglottis; C10.2-lateral wall of oropharynx; C10.3-posterior wall of oropharynx; C10.4-branchial cleft; C10.8-overlapping lesion of oropharynx; C10.9-oropharynx, NOS; C14.2-waldeyer ring. The HPV-unrelated (except oral tongue) sites were: C03.0-upper gum; C03.1-lower gum; C03.9-gum, NOS; C04.0-anterior floor of mouth; C04.1-lateral floor of mouth; C04.8-overlapping lesion of floor of mouth; C04.9-floor of mouth, NOS; C05.0-hard palate; C05.1-soft palate, NOS; C05.2-uvula; C05.8-overlapping lesion of palate; C05.9-palate, NOS; C06.0-cheek mucosa; C06.1-vestibule of mouth; C06.2-retromolar area; C06.8-overlapping lesion of other and unspecified mouth; C06.9-mouth, NOS; C12.9-pyriform sinus; C13.0-postcricoid region; C13.1-aryepiglottic fold, hypopharyngeal; C13.2-posterior wall of hypopharynx; C13.8-overlapping lesion of hypopharynx; C13.9-hypopharynx, NOS; C14.0-pharynx, NOS; C14.8-overlapping lesion of lip, oral cavity and pharynx. Lastly, the HPV-unrelated oral tongue sites were C02.0-dorsal surface of tongue, NOS; C02.1-border of tongue; C02.2-ventral surface of tongue, NOS; C02.3-anterior 2/3 of tongue, NOS; C02.8-overlapping lesion of tongue; C02.9-tongue, NOS.

Data from the original nine SEER registries (SEER 9) for cases diagnosed during the 34-year period from January 1, 1975, through December 31, 2008, were available for whites and blacks. These nine registries are located in the metropolitan areas of Atlanta, Detroit, San-Francisco-Oakland, and Seattle-Puget Sound and the states of Connecticut, Hawaii, Iowa, New Mexico, and Utah. For cases diagnosed during 1992–2008, data were available for the racial groups of whites, blacks, Asian/PIs, and AI/ANs and whether of Hispanic origin in the SEER 13, which includes the SEER 9 plus four additional areas, the California metropolitan areas of San Jose-Monterey and Los Angeles, rural Georgia, and Alaska Natives in Alaska. In the recent years 2004–2008, the SEER 9 and 13 registries accounted for about 10% and 14% of the US population, respectively [1]. All SEER registries annually meet the Gold Standard Registry Certification from the North American Association of Central Cancer Registries, Inc. for completeness (at least 95%), accuracy (<3% of cases identified through death certificates only), and timeliness of data. Age-adjusted incidence rates per 100,000 person-years of OCPC (using the 2000 US standard) were calculated for each subsite category (total, HPV-related, HPV-unrelated, and oral tongue) by sex and race/ethnicity using SEER*Stat [20]. All 95% confidence intervals (CIs) were estimated using the Tiwari modification [21]. Incidence rate ratios (IRRs) were calculated using females as the referent group.

Temporal trends in age-adjusted incidence rates using five- or six-year time periods 1975–79, 1980–85, 1986–91, 1992–97, 1998–2003, and 2004–2008 were plotted according to year of diagnosis by race/ethnicity, subsite category, and sex. Temporal trends in age-specific rates were plotted according to year of birth to facilitate visual comparison of rates. The year of birth was derived by subtracting age at diagnosis (midpoint of age group, i.e., 35 for age group 25–44, 50 for age group 45–54, 60 for age group 55–64, and 77.5 for age group 65+) from calendar year of diagnosis (midpoint of the calendar period); the cohorts are referred to by their approximate midyear of birth. Only rates based on at least 10 cases were presented. Temporal trends were plotted so that a slope of 10 degrees represented a change of 1% per year (i.e., 40 years on the horizontal axis is the same length as one logarithmic cycle on the vertical axis) [22]. Estimates of annual percent changes (EAPCs) were calculated for the period 1985–2008 using weighted least squares.

## 3. Results

There were 27,767 cases (18,573 men and 9,194 women) of OCPC diagnosed among whites and blacks during 1975–1991 in the SEER 9 and 43,679 cases (30,068 men and 13,611 women) among whites (non-Hispanic), blacks, Hispanics (white), Asian/PIs, and AI/ANs during 1992–2008 in the SEER 13 (Table 1). Total OCPC rates among men were 2–4 times those among women across each race/ethnicity and time period. Male/female (MF) IRRs for 1992–2008 SEER 13 were highest among blacks for each subsite (IRR = 2.60–4.32) and lowest among Asian/PIs (IRR = 2.39) for total, whites for HPV-related (IRR = 3.98) and HPV-unrelated (IRR = 2.16), and AI/ANs for oral tongue (IRR = 0.88, the only IRR not significantly higher than 1.0). The MF IRR for HPV-related cases (recent range 3.98–4.47) was higher than that for HPV-unrelated cases (recent range 2.16–3.37) in all instances except 1975–1991 SEER 9 blacks. The MF IRR for oral tongue (recent range 0.88–2.77) was smaller than that for other HPV-unrelated cases among each racial/ethnic group. Among whites and blacks in the SEER 9, the MF IRRs increased from 1975–1991 to 1992–2008 for HPV-related sites, but declined for HPV-unrelated sites and oral tongue.

**Figure 1:**
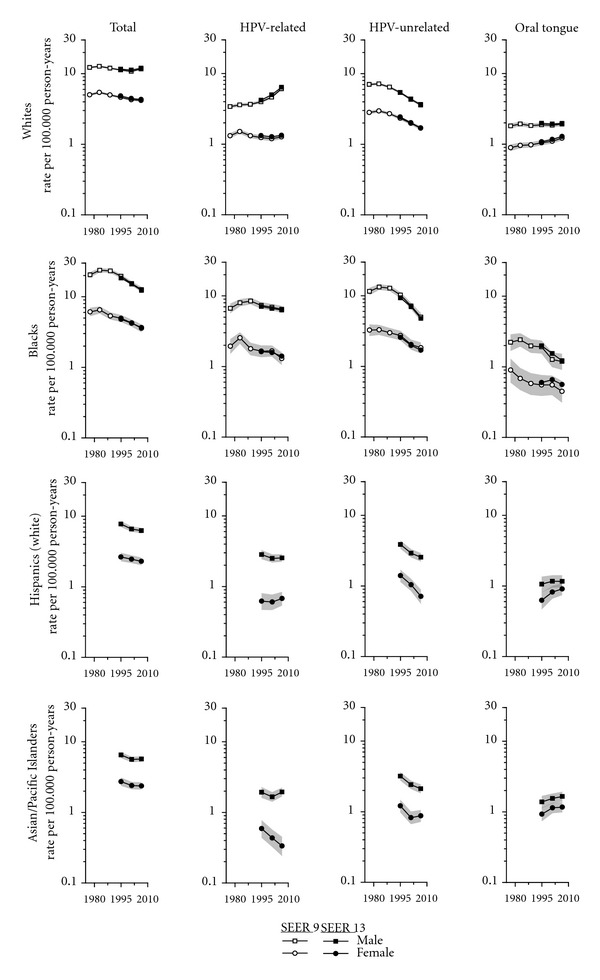
Trends in age-adjusted (2000 US standard) squamous cell carcinoma of the oral cavity and pharynx incidence rates by race/ethnicity, subsite, and gender for whites and blacks SEER 9 1975–1979 to 2004–2008 and for whites (non-Hispanic), blacks, Hispanics (white), and Asian/PIs SEER 13 1992–1997 to 2004–2008 according to year of diagnosis (excludes lip, salivary glands, and nasopharynx). Shaded bands portray 95% confidence intervals.

Trends in the age-adjusted OCPC incidence rates by race/ethnicity (whites (non-Hispanic), blacks, Hispanics (white), and Asian/PIs), subsite category, and gender in the SEER 13 during 1992–2008 are presented in Figure 1. For historical purposes, trend data for whites and blacks in the SEER 9 are also shown for 1975–2008. As seen in Figure 1, the two sets of rates in recent years were similar except for oral tongue among blacks where the rates were somewhat higher in the SEER 13 than the 9. Because the rates are based on larger numbers of cases and thus are more stable in the SEER 13 than the SEER 9, we will focus on them. Furthermore, it is increasingly important to distinguish non-Hispanics and Hispanics among whites because their rates are quite different, and the population proportions have been changing over time. Thus, we will use the term whites to refer to whites in the SEER 9 1975–1991 and white non-Hispanics in the SEER 13 1992–2008, blacks in the SEER 9 early years and SEER 13 recent years, and Hispanics for Hispanic whites and Asians for Asian/PIs in the SEER 13 recently. Among whites, total rates for men peaked at 12.7 per 100,000 person-years in 1980–1985 before declining gradually (11%) to 11.3 in 1998–2003 and then rose 5% to 12.0 in 2004–2008. Rates among women also peaked in 1980–1985 at 5.4 and declined more rapidly (20%) to 4.3 in 2004–2008. Among blacks, total rates for both men and women peaked during 1980–1985 and then declined dramatically until 2004–2008, by 48% from 23.7 to 12.3 among males and by 43% from 6.5 to 3.7 among females. During the recent 17-year period, total rates decreased significantly among all racial/ethnic/gender groups except white men.

HPV-related rates among white men rose 88% from 3.4 in 1975–1979 to 6.4 in 2004–2008, more rapidly in recent years than earlier, while HPV-unrelated rates declined 50% from 7.2 in 1980–1985 to 3.6 in 2004–2008 (Figure 1). Among the other race/sex groups, rates for HPV-related sites generally decreased or changed little. HPV-unrelated rates declined sharply among all race/sex groups, similar to white males. Rates decreased from 1980–1985 to 2004–2008 among white females by 43% from 3.0 to 1.7 and among blacks even more rapidly, by 64% among men from 13.3 to 4.8, and by 48% among females from 3.3 to 1.7. Rates also declined rapidly among Hispanics (men 34%, women 49%) and Asians (men 34%, women 28%) during 1992–2008. Oral tongue cancer rates among white men varied little over time, whereas rates in white women increased 44% from 0.9 in 1975–1979 to 1.3 in 2004–2008. Rates rose more rapidly among females than males among Hispanics (men 10%, women 45%) and Asians (men 19%, women 26%). In contrast, rates among blacks declined 50% for males (from 2.4 in 1980–1985 to 1.2 in 2004–2008) and 33% for females (from 0.9 in 1975–1979 to 0.6 in 2004–2008). The EAPCs from 1985 to 2008 among white males were +2.9% for HPV-related cancers and −3.1% for HPV-unrelated cancers, among white females −2.7% for HPV-unrelated cancers and +1.1% for oral tongue cancers, and among blacks for HPV-unrelated cancers −4.9% and −2.9% for males and females, respectively.

 The MF IRRs among whites doubled from 2.4 to 4.8 for HPV-related cancers declined modestly from 2.4 to 2.1 for HPV-unrelated cancers, and moderately from 2.0 to 1.5 for oral tongue cancers. Among blacks, the decrease in MF IRR for HPV-unrelated cancers was quite rapid, from 4.0 to 2.8.

**Figure 2:**
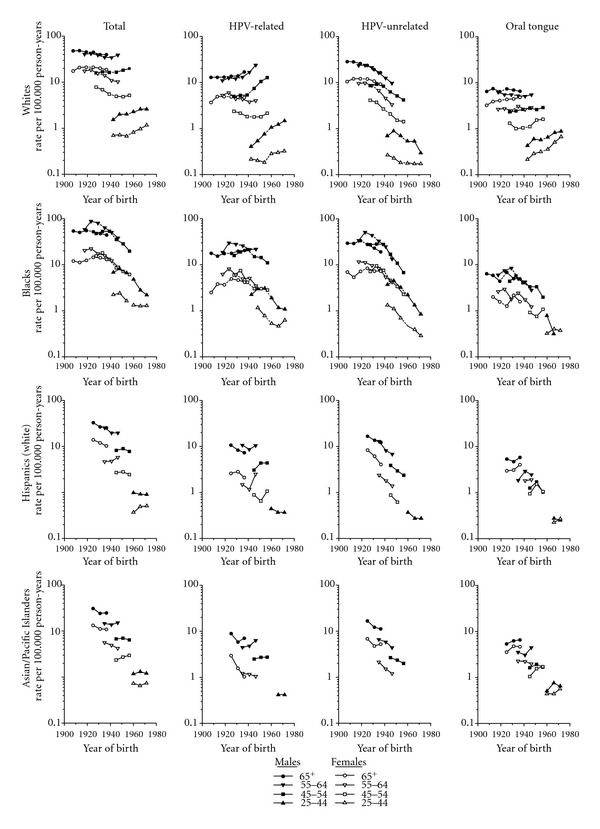
Trends in age-specific squamous cell carcinoma of the oral cavity and pharynx incidence rates by race/ethnicity, subsite, and gender for whites and blacks SEER 9 1975–1979 to 1986–1991 and for whites (non-Hispanic), blacks, Hispanics (white), and Asian/PIs SEER 13 1992–2008 according to year of birth (excludes lip, salivary glands, and nasopharynx) (rates age-adjusted within age group).

Among whites, total rates rose in the youngest age group among men and women due to substantial increases in successive birth cohorts for HPV-related cancers, more rapid among men than women, and oral tongue cancers, more rapid among women than men (Figure 2). The HPV-related rates among men aged 25–44 years more than tripled from 0.4 per 100,000 man-years in the 1942 birth cohort to 1.5 in the 1972 cohort, and rates among men aged 45–54 years more than doubled from 5.3 for the 1939 birth cohort to 12.8 for the 1956 birth cohort; rates also rose among men 55–64 from 12.5 for the 1923 cohort to 23.0 for the 1946 cohort. The EAPCs among men aged 25–64 each were >3%. Among women, HPV-related rates for the older age groups did not vary greatly in successive birth cohorts. For HPV-unrelated cancers, rates among white men declined 67% (from 0.9 to 0.3), 54% (from 9.2 to 4.2), 59% (from 23.9 to 9.7), and 42% (from 28.6 to 16.5) among those aged 25–44, 45–54, 55–64, and 65+ years, respectively; the EAPCs ranged between −3.1% and −4.0% among all but the oldest. Rates among women also declined 37% (from 0.27 to 0.17), 66% (from 4.1 to 1.4), 66% (from 9.8 to 3.3), and 24% (from 12.2 to 9.3), respectively. Oral tongue cancer rates among women aged 25–44 years more than tripled at +3.4% per year from 0.2 per 100,000 woman-years in the 1942 birth cohort to 0.7 in the 1972 cohort where rates approached those for young men, among whom rates increased 71% over the 30-year time period. Oral tongue rates also rose among women aged 45–54 years born since 1940 but not among men; rates for men or women in the older age groups did not change greatly.

Among blacks, total rates declined among cohorts born since at least 1930 reflecting the strong downward trends among men and women especially for HPV-unrelated sites (Figure 2). Black men aged 25–44 experienced the most rapid decrease in rates for HPV-unrelated sites across successive birth cohorts, 82% (from 4.5 to 0.8); and rates among males aged 45–54, 55–64, and 65+ years also declined impressively, 76% (from 28.3 to 6.8), 74% (from 51.1 to 13.4), and 43% (33.5 to 19.2), respectively. The corresponding EAPCs were −7.4% among those aged 25–44, and −6.8%, −5.5%, and −2.7% among the oldest. Rates decreased notably among women aged 25–44 (78%), 45–54 (76%), and 55–64 (64%), but not among women aged 65+. Oral tongue cancer rates among men generally declined across successive birth cohorts except for the older age groups where rates were relatively stable; in recent years among the youngest age group, the male predominance was no longer apparent. Of note, there was no suggestion of rapid increases in rates of HPV-related or oral tongue cancers among young blacks as seen among whites.

Among Hispanics and Asians, total OCPC trends were not consistent across the genders or age groups. However, rates of HPV-unrelated cancer generally declined across successive birth cohorts for both genders. Oral tongue cancer rates appeared to be converging among young men and women of both groups.

**Figure 3:**
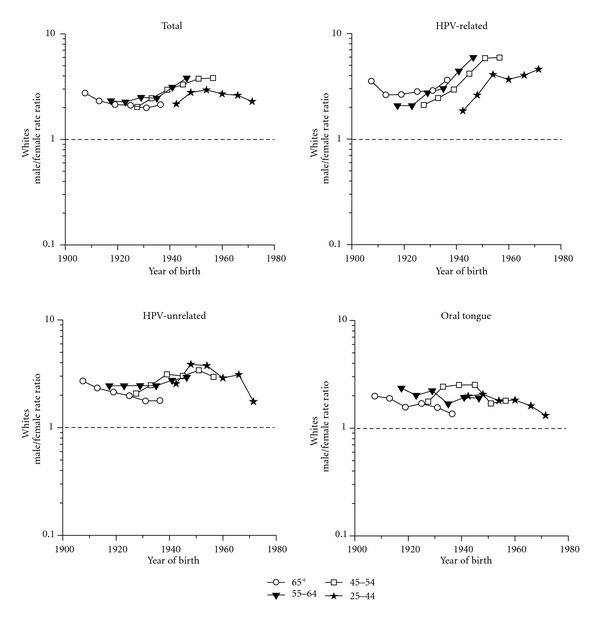
Trends in age-specific squamous cell carcinoma of the oral cavity and pharynx male/female incidence rate ratios among whites by subsite SEER 9 1975–1979 to 1986–1991 and whites (non-Hispanic) SEER 13 1992–2008 according to year of birth (excludes lip, salivary glands, and nasopharynx).

The MF IRRs for total among whites remained about 2 among those born prior to 1940, after which they rose to 2.9–3.8 among those born during the 1950s and then declined to 2.3 among those born around 1970 (Figure 3). MF IRRs for HPV-related cases were higher, hovering between 2 and 3 among those born prior to 1930, after which they rose to almost 6 among those aged 45–64 years born during the late 1940s–1950s; among those aged 25–44, the IRR rose from 1.9 among those born in the early 1940s to about 4 among those born since the mid-1950s. The MF IRRs for HPV-unrelated cases varied less by age group and year of birth, ranging between 2 and 3 among those born prior to the mid-1940s, rising to between 3 and 4 among those born during the 1950s and falling back to 3 or lower subsequently. Oral tongue cancer IRRs were all <3; among both the oldest and the youngest age groups, the IRRs decreased from about 2 to 1.3-1.4 over the study period.

## 4. Discussion

 In this study, we analyzed temporal trends of total OCP, presumed HPV-related, presumed HPV-unrelated, and oral tongue cancer among US men and women of white, black, Hispanic, Asian, and AI/AN ethnicity. Our study, which highlights changing patterns by gender, is based on the same categorization of anatomic subsites as presumed HPV-related or not used in two recent publications [11, 12], and we extend these findings by classifying cancer of the oral tongue separately from other HPV-unrelated sites, incorporating cases diagnosed through 2008, increasing the study population by including data from SEER 13, classifying whites as Hispanic or non-Hispanic, and presenting MF IRRs overall and by year of birth.

 Similar to other studies, we found overall OCPC incidence rates among men to be 2–4 times those among women of all race/ethnicities, and in recent decades, they varied from 4 or greater for HPV-related sites to 2-3 for HPV-unrelated sites, and 1–3 for oral tongue cancer. The MF IRR for HPV-related sites doubled among whites from 2.4 to 4.8 over the study period, due especially to rapidly rising rates of HPV-related OCPC among white men, particularly those born since 1940. Consistent decreases in the MF IRRs for HPV-unrelated sites were observed for whites (from 2.4 to 2.1) and blacks (from 4.0 to 2.8), due to rapidly declining rates especially among black men born since 1940. The IRRs for oral tongue cancer generally declined among all ethnic groups due especially to rising rates among younger white, Hispanic, and Asian women. Total OCPC trends varied markedly over the period 1985–2008. The age-adjusted rates for HPV-related cancers rose 3% per year among white males, for HPV-unrelated cancers declined 3% per year among whites of both genders and black females and 5% per year among black males, and for oral tongue cancer rose 1% per year among white females.

 The declines seen in HPV-unrelated cancer for all race/ethnic-sex groups are consistent with a pathway mainly driven by alcohol and tobacco (potentially related to p53 or other molecular alterations [23]) that parallel the reductions in US cigarette smoking prevalence [24] and may also reflect decreases in national per capita alcohol consumption, especially the use of hard liquor [25]. The steep declines in SCC of the OCP and racial/gender patterns seen here resemble the patterns for SCC of the esophagus, which also reflect the trends in cigarette smoking prevalence and alcohol consumption [26].

Among white males, the much smaller declines in the incidence of total OCPC appear to be driven by the rising incidence of HPV-related cancers, in contrast to the declines in HPV-unrelated cancers [27–29]. A number of studies have documented the increasing prevalence of HPV in oropharyngeal cancers during the past 30 years. In the US, the prevalence of HPV in oropharyngeal cancers rose from 33% during the 1980s to 70% in the 1990s and 82% during 2000–2004, based on 72 cases assayed in Colorado [10] and from 16% during the 1980s to 73% during the 2000s based on 271 cases from SEER tissue repositories in Hawaii, Iowa, and Los Angeles [13]. During the 2000s, several groups have reported HPV prevalence rates among oropharynx, tongue, and tonsil cases ranging from 63% to 92%, whereas the prevalence among other oral cancers has been 20% or less [30–32]. Thus, our assignment of presumed HPV status based on anatomic subsite certainly included non-HPV-related cases, more so during the earlier years compared to more recently. The incidence data suggest that the proportions also varied by gender, racial/ethnic group, and age.

HPV is a very common sexually transmitted infection in the United States [33]. Data from the 2003-2004 National Health and Nutrition Examination Survey (NHANES) revealed that the seroprevalence of any HPV types 6, 11, 16, or 18 (types in the quadrivalent vaccine) among persons aged 14–59 years was 22.4% overall, higher among females (32.5%) than males (12.1%), peaked at 42.0% among females aged 30–39 years and 18.0% among males aged 50–59 years, and was associated with lifetime number of sexual partners, especially among females [34]. About 90% of both females and males aged 25–44 ever had oral sex with an opposite-sex partner, based on data from the 2006–2008 National Survey of Family Growth (NSFG) [35]. The prevalence of oral infection with any of 37 HPV DNA types during 2009-2010 was 6.9% among those aged 14–69, significantly higher among men (10.1%) than women (3.6%), and increased with the number of sexual partners and cigarettes smoked per day, based on NHANES data [36].

Similar to other recent studies of oral tongue cancer [16, 37], we found increasing rates among young white adults aged 25–44 that were more rapid for women than for men, and some suggestion of similar patterns among blacks, Hispanics, and Asians. The causal pathway for oral tongue cancer in these patients is not known, but it does not appear to be related to use of alcohol or tobacco [16] or to the presence of HPV [16, 37]. Its unique age- and sex-specific incidence patterns suggest that it may have a different etiology from other OCPCs [37] including other oncogenic viruses, bacteria, and lifestyle and environmental factors.

 A limitation of our descriptive study is that HPV status is based on a categorization by anatomic subsite and thus likely resulted in some misclassification of actual HPV status. It has been suggested that HPV-related base of the tongue cancer may have been misclassified as oral tongue [11, 16]; however, the MF IRRs appear to be very different [37]. Cases without a particular site of the tongue specified (coded C02.9 = tongue, not otherwise specified) accounted for about 15% of oral tongue cases among whites during 1975–2008, and the proportion rose from 13% during 1975–1979 to 17% during 2004–2008. To the extent that some of these may have been HPV-related, then the observed increases in HPV-related cancers among white males have been underestimated. The observed increases in HPV-unrelated oral tongue cancer among white females may have been overstated; however, rates for each specific tongue subsite as well as tongue, NOS, all rose.

 In summary, our large numbers based on SEER 13 allowed us to plot race-, sex-, and age-specific incidence rates of OCPC by HPV category and year of birth and to carry out a detailed analysis of oral tongue cancers. The significant decreases in total OCPC incidence seen for most race/sex groups reflect reductions in smoking prevalence due to public health efforts aimed at cessation and lowering initiation rates as well as decreases in alcohol consumption. The increasing rates of cancer of the oral tongue especially among young men and women is of growing concern and suggest the need for dedicated research into the epidemiology of these understudied tumors; the narrowing of the gender differences apparent across all the racial/ethnic groups provides a clue worthy of pursuit. The much smaller decline in total OCPC incidence and recent increases among white men born since the mid-1940s appear related to the rapidly rising rates of HPV-related cancers and may reflect changes in sexual practices since the mid-1960s. The potential impact of HPV vaccination recently approved for both girls and boys on these trends is unknown, but changes are likely many years in the future. Dentists and physicians need to be aware of the notable and significant rise in rates of cancers of the oral tongue and HPV-related cancers of the oropharynx, especially among young men and women, and ensure that these new at-risk groups receive routine oral cancer screening.

## Figures and Tables

**Table 1:** Squamous cell carcinoma of the oral cavity and pharynx incidence by time period, race/ethnicity, sex, and subsite.

Year of diagnosis	SEER areas	Race/ethnicity	Sex	Total			HPV-related			HPV-unrelated			Oral tongue		
				Cases	rate/ratio	(95% CI)	Cases	rate/ratio	(95% CI)	Cases	rate/ratio	(95% CI)	Cases	rate/ratio	(95% CI)
1975–1991	9	All whites	Male Female	15,979 8,343	12.3 5.2	(12.1, 12.5) (5.1, 5.3)	4,682 2,227	3.6 1.4	(3.5, 3.7) (1.3, 1.4)	8,876 4,597	6.9 2.8	(6.7, 7.0) (2.8, 2.9)	2,421 1,519	1.9 0.9	(1.8, 1.9) (0.9, 1.0)

1975–1991	9	Blacks	Male Female	2,594 851	22.6 5.9	(21.7, 23.5) (5.5, 6.4)	901 300	7.8 2.1	(7.3, 8.4) (1.9, 2.3)	1,442 452	12.6 3.2	(12.0, 13.3) (2.9, 3.5)	251 99	2.2 0.7	(1.9, 2.5) (0.6, 0.9)

1992–2008	9	All whites	Male Female	18,539 8,533	11.2 4.4	(11.1, 11.4) (4.3, 4.4)	8,293 2,352	4.9 1.2	(4.8, 5.0) (1.2, 1.3)	7,155 4,009	4.4 2.0	(4.3, 4.5) (1.9, 2.1)	3,091 2,172	1.9 1.1	(1.8, 2.0) (1.1, 1.2)

1992–2008	9	Blacks	Male Female	2,711 934	15.5 4.2	(14.9, 16.2) (3.9, 4.5)	1,222 349	6.8 1.5	(6.4, 7.2) (1.4, 1.7)	1,245 469	7.3 2.2	(6.9, 7.7) (2.0, 2.4)	244 116	1.4 0.5	(1.2, 1.6) (0.4, 0.6)

1992–2008	13	Whites (non-Hispanic)	Male Female	22,657 10,586	11.6 4.5	(11.5, 11.8) (4.5, 4.6)	10,336 2,955	5.2 1.3	(5.1, 5.3) (1.3, 1.4)	8,553 4,936	4.5 2.1	(4.4, 4.6) (2.0, 2.1)	3,768 2,695	2.0 1.2	(1.9, 2.0) (1.1, 1.2)

1992–2008	13	Blacks	Male Female	3,690 1,311	15.1 4.2	(14.6, 15.6) (4.0, 4.4)	1,649 486	6.6 1.5	(6.3, 7.0) (1.4, 1.7)	1,663 631	6.9 2.1	(6.6, 7.3) (1.9, 2.2)	378 194	1.6 0.6	(1.4, 1.7) (0.5, 0.7)

1992–2008	13	Hispanics (white)	Male Female	1,895 791	6.8 2.4	(6.5, 7.1) (2.3, 2.6)	779 217	2.6 0.6	(2.4, 2.8) (0.6, 0.7)	803 303	3.0 1.0	(2.8, 3.3) (0.9, 1.1)	313 271	1.1 0.8	(1.0, 1.3) (0.7, 0.9)

1992–2008	13	Asian/PIs	Male Female	1,655 847	5.9 2.5	(5.6, 6.2) (2.3, 2.6)	528 153	1.8 0.4	(1.7, 2.0) (0.4, 0.5)	683 310	2.5 0.9	(2.3, 2.7) (0.8, 1.0)	444 384	1.5 1.1	(1.4, 1.7) (1.0, 1.2)

1992–2008	13	AI/ANs	Male Female	171 76	5.9 2.3	(5.0, 6.9) (1.8, 2.9)	72 18	2.2 0.5	(1.7, 2.8) (0.3, 0.8)	81 35	3.1 1.1	(2.4, 4.0) (0.8, 1.5)	18 23	0.6 0.7	(0.3, 0.9) (0.4, 1.0)

Male/female IRRs:															

1975–1991	9	All whites			2.38	(2.32, 2.44)		2.57	(2.45, 2.71)		2.42	(2.34, 2.51)		1.96	(1.84, 2.09)

1975–1991	9	Blacks			3.80	(3.52, 4.11)		3.73	(3.27, 4.25)		4.00	(3.60, 4.45)		3.11	(2.46, 3.93)

1992–2008	9	All whites			2.58	(2.68, 2.76)		3.99	(4.68, 5.19)		2.22	(1.73, 1.93)		1.69	(1.60, 1.85)

1992–2008	9	Blacks			3.70	(3.43, 3.99)		4.47	(3.97, 5.03)		3.37	(3.03, 3.75)		2.77	(2.22, 3.46)

1992–2008	13	Whites (non-Hispanic)			2.56	(2.50, 2.62)		3.98	(3.82, 4.14)		2.16	(2.09, 2.24)		1.67	(1.59, 1.75)

1992–2008	13	Blacks			3.61	(3.39, 3.84)		4.32	(3.91, 4.78)		3.37	(3.07, 3.69)		2.60	(2.19, 3.09)

1992–2008	13	Hispanics (white)			2.77	(2.55, 3.01)		4.11	(3.54, 4.78)		2.99	(2.62, 3.41)		1.43	(1.21, 1.68)

1992–2008	13	Asian/PIs			2.39	(2.20, 2.60)		4.21	(3.52, 5.04)		2.68	(2.35, 3.07)		1.41	(1.23, 1.62)

1992–2008	13	AI/ANs			2.59	(1.97, 3.39)		4.14	(2.47, 6.94)		2.85	(1.92, 4.24)		0.88	(0.47, 1.63)

Oral cavity and pharynx excludes lip, salivary glands, and nasopharynx.

HPV-related includes base of tongue, lingual tonsil, tonsil, oropharynx, and Waldeyer ring.

Oral tongue includes dorsal and ventral surfaces, border, anterior 2/3, overlapping lesions, and tongue not otherwise specified.

Rates per 100,000 person-years, age-adjusted using the US 2000 population standard; 95% CI = 95% confidence interval.

IRRs: incidence rate ratios, based on unrounded rates.

Asian/PIs: Asian/Pacific Islanders.

AI/ANs: American Indian/Alaska Natives.
